# Digital versus analogue chest drainage system in patients with primary spontaneous pneumothorax: a randomized controlled trial

**DOI:** 10.1186/s12890-020-1173-3

**Published:** 2020-05-11

**Authors:** Dieuwertje Ruigrok, Peter W. A. Kunst, Marielle M. J. Blacha, Ben Tomlow, Jacobine W. Herbrink, Eva J. Japenga, Wim Boersma, Paul Bresser, Ivo van der Lee, Kris Mooren

**Affiliations:** 1 Department of Pulmonary Medicine, Spaarne Gasthuis, PO Box 417 2000, AK Haarlem, the Netherlands; 2 Department of Pulmonary Medicine, OLVG, Amsterdam, The Netherlands; 3 Department of Pulmonary Medicine, NWZG, Alkmaar, The Netherlands

**Keywords:** Pneumothorax, primary spontaneous, Drainage, Chest tubes

## Abstract

**Background:**

Patients with a primary spontaneous pneumothorax (PSP) who are treated with chest tube drainage are traditionally connected to an analogue chest drainage system, containing a water seal and using a visual method of monitoring air leakage. Electronic systems with continuous digital monitoring of air leakage provide better insight into actual air leakage and changes in leakage over time, which may lead to a shorter length of hospital stay.

**Methods:**

We performed a randomized controlled trial comparing the digital with analogue system, with the aim of demonstrating that use of a digital drainage system in PSP leads to a shorter hospital stay.

**Results:**

In 102 patients enrolled with PSP we found no differences in total duration of chest tube drainage and hospital stay between the groups. However, in a post-hoc analysis, excluding 19 patients needing surgery due to prolonged air leakage, hospital stay was significantly shorter in the digital group (median 1 days, IQR 1–5 days) compared to the analogue group (median 3 days, IQR 2–5 days) (p 0.014). Treatment failure occurred in 3 patients in both groups; the rate of recurrence within 12 weeks was not significantly different between groups (16% in the digital group versus 8% in the analogue group, p 0.339).

**Conclusion:**

Length of hospital stay was not shorter in patients with PSP when applying a digital drainage system compared to an analogue drainage system. However, in the large subgroup of uncomplicated PSP, a significant reduction in duration of drainage and hospital stay was demonstrated with digital drainage. These findings suggest that digital drainage may be a practical alternative to manual aspiration in the management of PSP.

**Trial registration:**

Registered 22 September 2013 - Retrospectively registered, Trial NL4022 (NTR4195)

## Background

Primary spontaneous pneumothorax (PSP) is defined as the spontaneous occurrence of air in the pleural space in patients without clinically apparent lung disease [1]. The condition is associated with smoking [2, 3].

The management of PSP is based on two principles: first, the initial management of the pneumothorax with symptom control and re-expansion of the lung and secondly, reducing recurrence rate [4]. The recurrence rate is reported to be between 17 and 54% [5, 6], with the majority of recurrences in the first year after the primary event [7]. Recurrence rate can be reduced by surgical pleurectomy or thoracoscopic pleurodesis [8]. Initial management of PSP has been subject of ongoing debate [4, 8], with a trend towards a more conservative approach using manual aspiration and outpatient treatment [4, 6, 8–12]. Manual aspiration has been shown to be effective in approximately two-thirds of patients [8] and is as equally effective as chest tube drainage [8]. Since it is associated with reduced hospitalization or length of stay (LOS) [8], manual aspiration is recommended as the standard of care in international guidelines and recommendations [5, 13]. However, in daily practice, insertion of a small-bore pleural catheter or chest tube is still common practice in the Netherlands, probably due to practical reasons.

Until recently, the pleural catheter or chest tube was commonly connected to an analogue drainage system, in which air leakage is assessed by the observation of bubbling in the water seal. A major disadvantage of analogue systems is their inability to give accurate information about the quantity of air leakage and about air leak patterns. Uncertainty about air leakage might lead to unnecessary prolongation of hospital stay and additionally might increase the risk of recurrence, due to removing the tube before the pleural defect has healed [14].

Digital drainage systems provide quantitative information on air leakage (flow) in mL/min, both actual and historical, thereby providing information on air leakage patterns [15]. In thoracic surgical patients these drainage systems have been shown to reduce duration of hospital stay, compared to analogue drainage systems [16]. Digital monitoring of air leakage patterns might be useful in predicting prolonged air leakage after pulmonary resection, thereby providing a window-of opportunity for early intervention [17].

Whether digital drainage of PSP affects duration of hospital stay has yet to be established. The aim of the study was to investigate whether the use of a digital drainage system in PSP leads to a shorter length of (hospital) stay compared to the use of an analogue drainage system.

## Methods

### Study design

The Dutch Pneumothorax Study (DPS) was a multicentre, randomized controlled trial, carried out in five Dutch hospitals (4 large teaching hospitals and 1 regional hospital) between 2012 and 2017. The study compared the efficacy (LOS) and safety (recurrence rate of pneumothorax within twelve weeks) of a digital drainage system compared to conventional analogue drainage systems in patients with PSP and a need for clinical treatment with chest drainage. Blinding was not possible due to the nature of the study. This study, within the scope of the Medical Research Involving Human Subjects Act, was approved by the Medical Ethics Review Committee of the Academic Medical Centre/University of Amsterdam (NL36778.018.1). The trial was registered in the Netherlands Trial Register https://www.trialregister.nl/trial/4022). This was an investigator-initiated study; after the study protocol was written, Medela Benelux BV provided the participating centres with the Thopaz digital drainage system for the duration of the study. The company was not involved in study design, trial execution or data analysis.

### Study subjects

Patients with PSP who were treated with chest tube drainage were asked to participate in the study. Those with recurrent pneumothorax were referred to surgery directly and were therefore not included. Study subjects had to be 18 years or older. Patients with respiratory failure, intensive care unit admission or with uncontrolled bleeding tendency were excluded from the study.

### Procedures

Patients were randomized between the analogue and digital drainage system. Randomization was performed centrally using a web-based program (Research Manager©). The digital drainage system consisted of Thopaz (Medela Healthcare, Switzerland); analogue drainage systems were not predefined, locally available systems per participating centre were used.

**Fig. 1:**
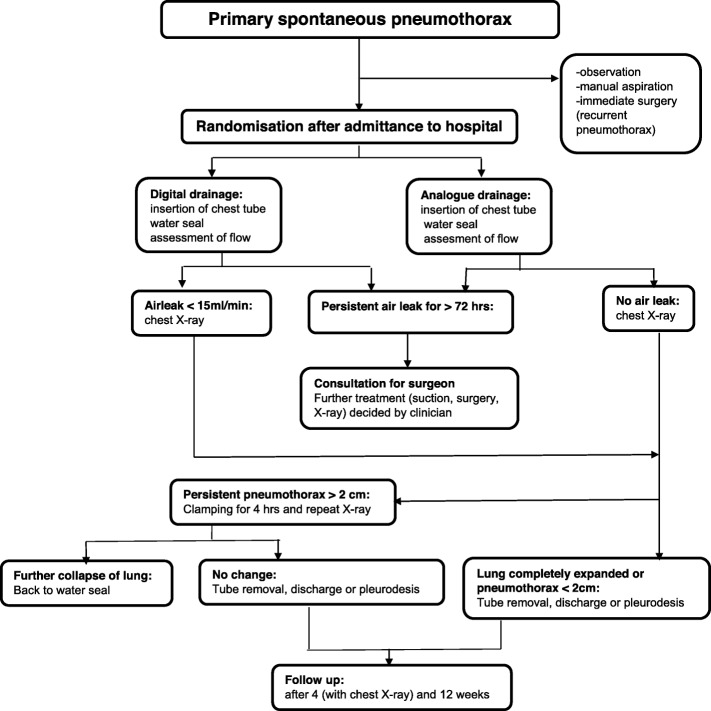
Study flow chart

Choice of chest tube size and insertion site was left to the treating clinician. In both groups, the drainage system was initially put on water seal [18, 19]. Air leakage was assessed three times daily by professional medical staff. If no air leakage was visible (analogue group), or if the air leak was below 15 mL/min (digital group) [20], a chest X-ray was performed (Fig. 1). If the longest distance between lung and chest wall was less than 2 cm on the chest X-ray [21], the chest tube was removed and the patient was discharged. If the chest X-ray showed a pneumothorax larger than 2 cm (both groups), the tube was clamped for 4 h and the chest X-ray was repeated. If the pneumothorax did not increase, the tube was removed and the patient was discharged. If the chest X-ray showed an increase in pneumothorax size, the clamp was removed and the water seal reapplied [22, 23]. If an air leak existed for more than 72 h, a thoracic surgeon was consulted and all further treatment strategies (such as applying suction) were left to the treating clinician. All medical decisions were recorded in a web-based case report form (CRF). All patients had a follow-up visit with chest X-ray scheduled at the outpatient clinic 4 weeks after discharge. After twelve weeks, patients were asked by means of a telephone call whether they had sought medical care for symptoms of recurrence of pneumothorax.

The following variables were recorded at baseline: self-reported smoking status, number of pack.

years, previous medical history, length and weight.

### Statistical analysis

Primary outcome of this randomized controlled trial was efficacy of a digital drainage system in patients with a primary spontaneous pneumothorax, defined as a reduction in LOS (duration of hospital stay) compared to patients treated with drainage using an analogue drainage system. Secondary objectives were treatment failure, defined by clinically relevant recurrence of pneumothorax within 1 week of discharge, or a recurrence of pneumothorax within twelve weeks leading to a medical intervention such as manual aspiration, drainage, thoracoscopy or surgery.

To obtain a reduction of 20% in length of hospital stay (with a standard deviation of 20%, α = 0.05 and β = 0.9) a sample size of 86 subjects in each treatment arm was needed. Considering a possible dropout rate of 10%, a target inclusion rate of 190 patients was set.

Data were interpreted according to the intention-to-treat principle. Crossover to another drainage system was allowed as were any off-protocol decisions made by the treating physician based on best medical judgement.

Data are presented as mean (standard deviation, SD) or median (interquartile range, IQR), depending on the number of patients and distribution of values. Normal distribution was tested by using D’Agostino-Pearson omnibus normality test. Differences in baseline characteristics, efficacy and safety were tested using unpaired T-test/Mann Whitney test or Chi-square/Fisher exact test where appropriate. Values of *p* < 0.05 were considered to reflect statistical significance. Statistics were performed using GraphPad Prism version 7.0b (GraphPad Software, La Jolla, California, USA).

## Results

From October 2012 until September 2017 a total of 102 patients with a spontaneous pneumothorax were included: in 50 patients an analogue drainage system was used, and in 52 patients the digital drainage system was used. The study was terminated before the target of 190 patients was reached due to slower inclusion than expected.

Both groups had the same demography except for BMI (Table 1). Males were predominant in both groups (80–88%). The majority of patients had a first episode of spontaneous pneumothorax. In retrospect 6 cases of secondary spontaneous pneumothorax were included (4 in the analogue group and 2 in the digital group); these patients with secondary spontaneous pneumothorax were diagnosed with an underlying pulmonary condition after their hospital admission with pneumothorax.

**Table 1:** Baseline characteristics

Variable	Analogue drainage ***N*** = 50	Digital drainage ***N*** = 52	***P***-value
Age (years)	32 (range 18–86)	33 (range 18–89)	0.869
Male (n, %)	40 (80%)	46 (88%)	0.284
Height (cm)	180 (170–188)	180 (177–186)	0.386
BMI (kg/m^2^)	20.9 (18.5–23.6)	22.3 (20.0–24.2)	0.035*
Left sided pneumothorax (n, %)	21 (42%)	21 (40%)	0.868
Current/former smokers (n, %)	38 (76%)	45 (87%)	0.208
First episode of pneumothorax (n, %)	47 (94%)	46 (88%)	0.488
Secondary spontaneous pneumothorax (n, %)	4 (8%)	2 (4%)	0.432

Data presented as median (IQR) or number of patients (%) unless otherwise stated. BMI: body mass index. Statistical tests used: Mann Whitney test, Fisher’s exact test, Chi-square test. Statistical significance indicated with an *

Crossover to the digital drainage system occurred 4 times in the analogue group, mainly due to the reduced mobility with suction drainage using the analogue system. Crossover occurred only once in the digital group, after surgery due to logistic reasons.

### Primary outcome

In the whole group of patients, total duration of chest tube drainage and LOS did not differ between the two groups (Table 2). However, 19 patients underwent surgery because of prolonged air leakage, 6 in the analogue group and 13 in the digital group (p 0.127). After excluding these subjects with a complicated course, both the duration of chest tube drainage and LOS were significantly shorter in the patients randomized to digital drainage (*n* = 39) compared to analogue drainage (*n* = 44): median 1 (IQR 1–3) versus 3 (IQR 2–5) days (p 0.024) and median 1 (IQR 1–5) versus 3 (IQR 2–5) days (p 0.014) respectively (Table 3).

**Table 2:** primary and secondary outcomes

Variable	Analogue ***N*** = 50	Digital ***N*** = 52	***P***-value
Total duration chest tube drainage (days)	3 (2–5)	2 (1–12)	0.488
LOS (days)	3 (2–6)	2.5 (1–14)	0.640
Number of patients undergoing surgery (n, %)	6 (12%)	13 (25%)	0.127
Number of chest X-ray during admission	2.5 (1–4)	2 (1–3)	0.158
Treatment failure (recurrence within one week) (n, %)	3 (6%)	3 (6%)	> 0.999
Recurrence within 12 weeks follow-up (n, %) (excluding those with treatment failure)	7 (16%) (missing follow-up in *n* = 3)	4 (8%)	0.339

Data presented as median (IQR) or number of patients (%). LOS: length of stay. Statistical tests used: Mann Whitney test, Fisher’s exact test

**Table 3:** Primary and secondary outcomes in subgroup with “uncomplicated” pneumothorax

Variable	Analogue ***N*** = 44	Digital ***N*** = 39	***P***-value
Total duration chest tube drainage (days)	3 (2–5)	1 (1–3)	0.024*
LOS (days)	3 (2–5)	1 (1–5)	0.014*
Number of chest x-ray during admission	2 (1–3)	1 (1–2)	0.017*
Treatment failure (recurrence within one week) (n, %)	3 (7%)	2 (5%)	> 0.999
Recurrence within 12 weeks follow-up (n, %) (excluding those with treatment failure)	6 (16%) (missing follow-up in *n* = 3)	2 (5%)	0.262

Data presented as median (IQR) or number of patients (%). LOS: length of stay. Statistical tests used: Mann Whitney test, Fisher’s exact test. Statistical significance indicated with an *

### Secondary outcomes

Clinically relevant pneumothorax within 1 week of discharge occurred in 6% (*n* = 3) in both groups (Table 2). Recurrence, arbitrarily defined as a clinically relevant pneumothorax within 12 weeks (excluding those with recurrence within 1 week of discharge) occurred in 7 (16%) patients in the analogue group versus 4 (8%) patients in the digital drainage group (p 0.339) (Table 2). In the analogue group, three patients had a recurrence or increase of pneumothorax size at the time of the scheduled follow-up visit after 4 weeks; in one of them this was not clinically relevant and spontaneous resolution was observed; two patients underwent surgical intervention as a result of the recurrent pneumothorax. In the digital group, only one patient had an increase in pneumothorax size at the time of the scheduled follow-up 4 weeks after discharge, which was not clinically relevant and had a spontaneous resolution.

## Discussion

To our knowledge, this trial is the largest multicentre randomized controlled clinical trial concerning PSP that has been conducted to date. Our reason for performing this study was the introduction of digital drainage systems, which potentially have an advantage in treating PSP. Digital drainage systems have been extensively evaluated in patients who have undergone thoracic surgery [22, 23], but not in PSP. In PSP, there are recent data that digital drainage might identify patients who benefit from early referral to surgery, but further data collection is ongoing [24].

In this randomized controlled trial comparing digital and analogue chest tube drainage systems in primary spontaneous pneumothorax patients, total duration of chest tube drainage and LOS were not different between both groups. However, after excluding those patients with prolonged air leak (i.e. those undergoing surgery, medical thoracoscopy and/or pleurodesis), duration of chest tube drainage and LOS were significantly shorter (median 1 vs 3 days) in patients randomized to digital drainage compared to analogue drainage. The use of digital drainage is safe when compared to the analogue system, since there was no significant difference in treatment failure between the groups.

For the whole group of PSP patients LOS was comparable between the analogue and the digital drainage group. However, the large majority of patients (83/102) was treated with drainage only (i.e. without thoracoscopy, surgery or pleurodesis); in these patients we observed a highly significant difference in LOS. Therefore, in the subgroup analysis of patients without persistent air leak, which we call uncomplicated PSP, digital drainage was shown to shorten LOS in the patients studied here. The fact that we did not find a difference in hospital stay between the two complete groups may be explained by the large effect exerted by those with a prolonged air leak, on a relatively small study.

population.

According to current guidelines, manual aspiration is regarded as first choice therapy in PSP. Based on our subgroup analysis, digital drainage possibly has an advantage over analogue systems in uncomplicated PSP. Digital drainage might be considered a practical alternative for manual aspiration: after insertion of a chest tube, the clinician connects it to a digital device instead of evacuating air manually. As soon as the digital device indicates that there is less than 15 ml air flow per minute and the chest X-ray shows no more than a small pneumothorax, removal of the chest tube appears to be safe. According to our study data, the chance of treatment failure is much lower than the percentages found in trials on manual aspiration (manual aspiration success rates range from 30 to 80% [11], although the definition of success rate varies considerably between the various studies). One could hypothesize that if we set the air leak threshold lower than 15 ml/min, the failure rate could drop even further. It is noteworthy that there is no consensus between clinicians on airflow threshold values for chest tube removal [20].

Unfortunately, due to the large variability in clinical management despite the trial algorithm, we cannot make meaningful statements regarding the usefulness of performing a chest X-ray in the absence of air leakage or clamping the chest tube in the absence of air leakage.

A noteworthy finding is the follow-up chest X-ray and outpatient clinic appointment, which at present is common practice. It is questionable whether this is necessary, as only 4 of all study subjects (4%) had a recurrence of pneumothorax at this scheduled follow-up.

An interesting aspect of digital drainage, that was not been evaluated in the current study, is air leakage patterns, as suggested by Takamochi et al. [17]. Patients who have a high, non-declining flow rate are likely to have a persistent pleural defect and to benefit from early referral for surgery. Patients with a declining flow rate are likely to benefit from a conservative approach. A recent study by Hallifax et al. indeed demonstrated that digital air leak measurements early in the treatment course potentially predicts future treatment failure [24].

Another potential subject for research is outpatient treatment of PSP with digital drainage systems. Digital drainage could facilitate home treatment since flow rate can easily be assessed and reported by the patient. It may also be of interest to compare these management strategies with manual aspiration in future studies.

In our view, the main strength of this study is that it is a ‘real life’ study in a common but underresearched condition. It helps us to define how to implement digital drainage systems in our daily clinical practice, and to re-evaluate the way we approach patients with PSP. On the other hand, our study has some important limitations. In PSP, when chest tube placement is indicated, it is usually placed shortly (several hours) after the diagnosis has been made – frequently out of hours. In the five participating hospitals, patients were seen both by doctors working for the pulmonary division as emergency doctors, for whom randomization was often too time-consuming. This led to missed inclusions and a slow inclusion rate. Therefore, the study was terminated before the target sample size was reached. The missed inclusions may have led to a selection bias. Thirdly, the use of different types of chest tubes (including different chest tube diameters) might have influenced the results.

In conclusion, although a difference could not be shown for the whole group of PSP patients, a significant reduction in length of hospital stay was shown in the patients with uncomplicated PSP (not needing surgery due to prolonged air leakage) when using a digital drainage system.

## Conclusions

Length of hospital stay was not shorter in patients with PSP when applying a digital drainage system compared to an analogue drainage system. However, in the large subgroup of uncomplicated PSP, a significant reduction in duration of drainage and hospital stay was demonstrated with digital drainage. Whether digital drainage has an advantage over manual aspiration, and whether digital drainage has the potential to predict which patients benefit from early referral to surgery, is an important subject for further research.

## Data Availability

The complete study protocol, datasets used and/or analysed during the current study are available from the corresponding author on request.

## Abbreviations

PSP: Primary spontaneous pneumothorax
LOS: Length of stay
DPS: Dutch Pneumothorax Study
CRF: Case report form
SD: Standard deviation
IQR: Interquartile range
BMI: Body mass index
